# Complete Remission of Multiple Brain Metastases in a Patient with *EGFR*-Mutated Non-Small-Cell Lung Cancer Treated with First-Line Osimertinib without Radiotherapy

**DOI:** 10.1155/2020/9076168

**Published:** 2020-03-19

**Authors:** Koken Ameku, Mariko Higa

**Affiliations:** ^1^Department of Respiratory Medicine, Okinawa Prefectural Nanbu Medical Center & Children's Medical Center, Okinawa, Japan; ^2^Department of Respiratory Medicine, Okinawa Prefectural Yaeyama Hospital, Okinawa, Japan

## Abstract

Osimertinib has demonstrated efficacy against stable or asymptomatic central nervous system (CNS) metastases of epidermal growth factor receptor (*EGFR*) mutation-positive non-small-cell lung cancer (NSCLC) in phase 2 and 3 clinical trials that allowed prior CNS radiotherapy. However, the efficacy of osimertinib only or the optimal treatment combination or sequence of radiotherapy has not been investigated. A 74-year-old woman diagnosed with T4N1M1c Stage IVB lung adenocarcinoma with *EGFR* mutation presented with a left upper lobe mass and multiple bilateral lung metastases. A total of more than 20 asymptomatic multiple brain metastases with a maximum diameter of 12 mm were diagnosed simultaneously. Osimertinib was administered as first-line treatment. Whole brain radiotherapy was deferred because she had no neurological symptoms. After 5 weeks, the multiple brain metastases disappeared completely, together with the response in the lung lesions. This case demonstrated that first-line treatment with osimertinib could even achieve complete remission of multiple brain metastases comprising as many as twenty lesions of *EGFR*-mutated NSCLC without radiation therapy. Radiation therapy for brain metastases can be deferred or even withheld. A new treatment strategy for *EGFR* mutated NSCLC with CNS metastases should be investigated using osimertinib, especially regarding optimal combination or sequence of radiotherapy.

## 1. Introduction

Brain metastasis often occurs as a serious complication in non-small-cell lung cancer (NSCLC) patients and leads to a deterioration in the quality of life (QOL) and reduced overall survival (OS) [1]. Almost 25% of patients with epidermal growth factor receptor (*EGFR*) mutation-positive NSCLC present with accompanying central nervous system (CNS) metastases at the first diagnosis. Moreover, despite treatment with EGFR tyrosine kinase inhibitors (TKIs), the incidence of CNS metastases increases to more than 45% at 3 years after diagnosis [2].

The efficacy of first-generation EGFR-TKIs against CNS metastatic lesions of *EGFR*-mutated NSCLC has been shown in several small phase 2 trials [3–5]. This is attributable to their ability to cross the blood-brain barrier (BBB) probably due to their low molecular weight [6]. However, their concentration in the cerebrospinal fluid is much lower than in the blood, which leads to frequent CNS failure after achieving systemic clinical benefit mainly due to pharmacokinetic limitations following acquisition of the T790M resistant mutation [7]. There is limited clinical evidence including the above regarding the treatment of *EGFR*-mutated NSCLC with synchronous CNS metastases, because these patients have generally been excluded from clinical trials.

Osimertinib is an oral CNS-active, third-generation irreversible EGFR-TKI that selectively inhibits both EGFR-TKI-sensitising and EGFR T790M-resistance mutations. A preclinical model showed that osimertinib is highly distributed in the mouse and nonhuman primate brain with greater exposure than gefitinib or erlotinib, indicating the CNS penetration of osimertinib [8]. In the phase 2 AURA extension [9], phase 2 AURA2 [10], and phase 3 AURA3 [11] trials of a T790M-positive population and in phase 3 FLAURA trial [12] of an untreated population, patients with stable, asymptomatic CNS metastases, with or without prior CNS treatment, were eligible for enrolment. In the preplanned subgroup analysis, osimertinib showed robust systemic and intracranial efficacy against *EGFR*-mutated NSCLC patients with CNS metastases in both the untreated population and the population with acquired T790M resistance mutation after prior EGFR-TKI treatment [13–15]. Patients with measurable and/or nonmeasurable CNS metastases on available brain scans were included in the CNS full analysis set (cFAS). The CNS evaluable-for-response (cEFR) set included only patients with more than one measurable CNS lesion with the longest diameter ≥ 10 mm or at least two times the slice thickness or reconstruction interval. In the CNS cEFR set, CNS objective response rates were reported as 54%, 70%, and 91% and the CNS disease control rates as 92%, 93%, and 95%, in the pooled data from two phase 2 trials (AURA extension and AURA2), AURA3 trial, and FLAURA trial, respectively. In the CNS cFAS, the median CNS progression-free survival (PFS) period was reported as 11.7 months and not reached (95% confidence interval (CI), 16.5 months to not calculable), and the median CNS duration of response was reported as 8.9 months and not reached (95% CI, 11.9 months to not calculable), in the AURA 3 and FLAURA trials, respectively. CNS response was obtained regardless of prior brain radiotherapy [13–15].

Treatment options other than EGFR-TKIs are surgical resection or stereotactic radiosurgery (SRS) for limited numbers of brain metastases and whole-brain radiotherapy (WBRT) for multiple brain metastases. However, the optimal treatment combination or sequence of radiotherapy with EGFR-TKIs of all generations has not been clearly studied [16].

To date, limited studies have assessed the efficacy of osimertinib against CNS metastasis, including the efficacy of osimertinib only, or the optimal combination or sequence of radiotherapy. We report the first single case that described complete remission of multiple brain metastatic lesions of *EGFR*-mutated NSCLC treated with first-line osimertinib without radiotherapy.

## 2. Case Presentation

**Figure 1:**
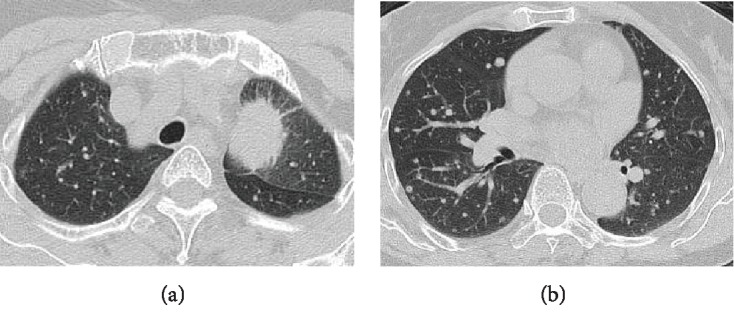
The patient was diagnosed with T4N1M1c Stage IVB lung adenocarcinoma with *EGFR* exon 19 deletion mutation presenting as (a) 40 mm wide shadows in the upper lobe of the left lung and (b) multiple bilateral small nodules in the entire lung field.

**Figure 2:**
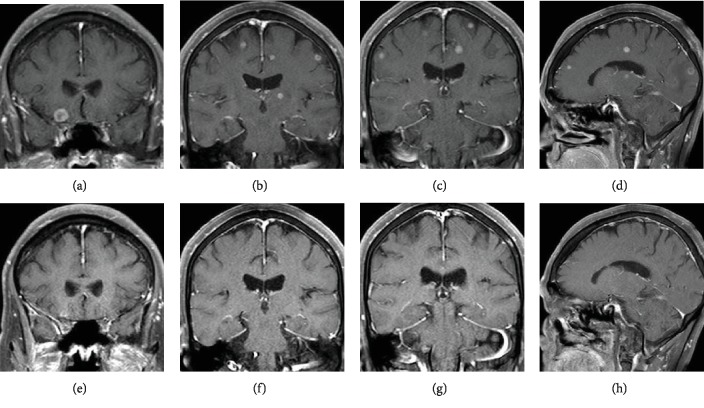
Multiple asymptomatic brain metastatic lesions (more than 20 in total) with a maximum diameter of 12 mm were detected by gadolinium-enhanced MRI at the first diagnosis (a–d). After 5 weeks, the multiple brain metastases had disappeared completely on contrast-enhanced brain MRI (e–h). Complete CNS response was confirmed.

A 74-year-old woman with no history of smoking underwent a computed tomography scan of the chest and abdomen on account of an elevated blood carcinoembryonic antigen level. The computed tomography scan revealed a 40 mm shadow in the upper lobe of the left lung and multiple bilateral small nodules in the entire lung field (Figures 1(a) and 1(b)). She was diagnosed with T4N1M1c Stage IVB lung adenocarcinoma with *EGFR* exon 19 deletion mutation. More than 20 multiple asymptomatic brain metastases with a maximum diameter of 12 mm were detected via gadolinium-enhanced brain magnetic resonance imaging (MRI) (Figures 2(a)–2(d)). Osimertinib 80 mg once daily was administered as first-line treatment. WBRT was deferred because she had neither physical complaints nor neurological symptoms and assessed as performance status 0. After two weeks, the bilateral abnormal small lung nodule shadow had diminished on a chest radiograph. After 5 weeks, the multiple brain metastases had also disappeared completely on enhanced brain MRI (Figures 2(e)–2(h)). Complete CNS response was confirmed. Osimertinib has been continued, and complete CNS response has been maintained after 4 months of treatment. She has been well with neither symptoms nor adverse events.

## 3. Discussion

This case illustrates several important clinical findings. First, osimertinib can result in complete remission of multiple brain metastasis in patients with as many as twenty *EGFR*-mutated NSCLC lesions without radiation therapy when used as first-line treatment. This was also found in the phase 3 FLAURA trial that used osimertinib as first-line treatment for *EGFR*-mutated NSCLC patients [15]. In the trial, patients with asymptomatic or stable CNS metastases, with or without prior CNS treatment, were included; patients with symptomatic or unstable CNS metastases were only included if stable for ≥2 weeks after completion of definitive therapy and corticosteroids. Among patients with CNS metastases, 25% in the osimertinib arm and 24% in the standard EGFR-TKI arm were treated with prior brain radiotherapy within 6 months of participation in the study. The preplanned exploratory analysis demonstrated a nominally statistically significant and clinically meaningful improvement in CNS PFS with osimertinib over standard EGFR-TKIs (gefitinib or erlotinib) in the subgroup of patients with CNS metastases (median PFS: not reached vs. 13.9 months, hazard ratio (HR) = 0.48; *p* = 0.014), with a 52% reduction in the risk of CNS progression. The objective CNS response rates were 66% (cFAS) and 91% (cEFR) in the osimertinib arm and 43% (cFAS) and 68% (cEFR) in the standard EGFR-TKI arm. The complete CNS response rates were 41% (cFAS; *n* = 25) and 23% (cEFR; *n* = 5) in the osimertinib arm and 24% (cFAS; *n* = 16) and 0% (cEFR) in the standard EGFR-TKI arm. Complete CNS response was achieved without prior brain radiotherapy in all five patients in the cEFR set of the osimertinib arm. Complete CNS response was observed in both the nonmeasurable (cFAS) and measurable (cEFR) CNS disease groups. The CNS benefit of osimertinib was acknowledged irrespective of prior brain radiotherapy. The maximum diameter of the brain lesion, in this case, was 12 mm, which was categorised as measurable CNS metastasis. It is suggested that complete CNS response can be obtained in approximately one-fourth of cases regardless of the size, number of metastatic lesions, and radiotherapy status. It is also suggested that a more complete response can be achieved in smaller lesions, as observed in the cFAS. Osimertinib can be considered to have greater benefit than standard EGFR-TKI, especially in patients presenting with brain lesions at diagnosis that occur in almost one-fourth of *EGFR*-mutated NSCLC cases. To the best of our knowledge, this is the first single case report that described complete remission of as many as twenty metastatic brain lesions of *EGFR*-mutated NSCLC with first-line osimertinib without radiotherapy.

Second, radiotherapy for brain metastases can be deferred or even withheld especially when osimertinib is used as first-line treatment, because complete CNS remission can occasionally be achieved as early as one month even with multiple lesions as many as twenty. The median time to CNS response was 5 weeks in this case and 6 weeks in the osimertinib arm of the FLAURA trial [15]. A treatment strategy involving upfront osimertinib followed by radiotherapy, if needed, can be considered. Especially in cases of complete remission, radiotherapy could be withheld. Thus, the benefits and risks of combining radiotherapy with EGFR-TKIs should be considered.

Regarding benefits, a combination of EGFR-TKIs and radiotherapy has been hypothesised to result in radiosensitisation and increased permeability of the BBB to EGFR-TKIs [16, 17]. Many meta-analyses and retrospective studies regarding the combination of EGFR-TKIs with radiotherapy have yielded conflicting results. Some studies have reported the superior efficacy and survival benefit of upfront radiotherapy or radiotherapy plus EGFR-TKIs over EGFR-TKIs alone [18–22]. However, others have reported no survival benefit [23–26]. Thus, the optimal treatment combination or sequence for brain metastasis remains controversial [16]. Besides, these studies had many limitations, owing to the retrospective analyses, such that the majority did not examine the late effects of radiotherapy on neurocognitive function. The results of these studies do not apply to osimertinib, because the first and second generation EGFR-TKIs, gefitinib, erlotinib, afatinib, and icotinib, were used in these studies. Although there is no study that reported the benefit of combining radiotherapy with osimertinib at the moment, the consistent lower incidence of CNS progression events with osimertinib compared with standard EGFR-TKIs in the subgroup of patients with known or treated CNS metastasis at study entry (20% vs. 39%), without known or treated CNS metastasis at study entry (3% vs. 7%), and the overall FLAURA population (6% vs. 15%) may make it possible to defer radiotherapy [12].

By way of comparison of radiotherapy and EGFR-TKIs, only one phase 3 trial (the BRAIN study) compared the efficacy and safety of WBRT plus chemotherapy with the EGFR-TKI icotinib in *EGFR*-mutated NSCLC patients with multiple symptomatic or asymptomatic (more than three) brain metastases. The study reported a significantly longer median CNS PFS (10.0 months vs. 4.8 months; HR = 0.56; *p* = 0.014) and numerically fewer treatment-related adverse events of grade 3 or worse (8% vs. 38%) with icotinib compared with WBRT plus chemotherapy as first-line treatment. Both groups could cross over to each other after progression, and there were no significant differences in median OS (18.0 months vs. 20.5 months; HR = 0.93; *p* = 0.734) and time to increased brain metastases symptoms (18.0 months vs. 19.0 months; HR = 0.75; *p* = 0.284) [27]. This study demonstrates the relatively small benefit of WBRT compared with icotinib as first-line treatment and that icotinib resulted in comparable OS even when WBRT was deferred. Although no clinical trials have directly compared osimertinib with radiotherapy, the results of this study imply that osimertinib has a similar superior efficacy to WBRT plus chemotherapy as icotinib, considering the superior efficacy of osimertinib compared to standard EGFR-TKIs [15].

From the viewpoint of risks, extended survival in *EGFR*-mutated NSCLC emphasises the importance of balancing both OS and QOL which can be affected by the subacute and late neurologic complications of radiotherapy [1, 17, 28]. As for WBRT, unacceptable levels of toxicity of the combination of WBRT plus SRS and erlotinib [29] and worse health-related QOL [30] were demonstrated in two phase 3 trials. SRS can be a substitute for WBRT, as a recent trial (JLGK0901) demonstrated noninferior OS with SRS without WBRT in patients with five to ten brain metastases when compared to patients with two to four metastases (median OS; 10.8 months vs. 10.8 months; HR = 0.97; *p* noninferiority < 0.0001) [31]. However, even in SRS, the rate of leucoencephalopathy is suggested to increase up to 84% in 4 years [32].

This case indicates that even when multiple CNS lesions are as many as twenty, WBRT can be deferred while expecting the remission of a large proportion of the lesions. Deferral and even withholding WBRT and performing SRS as needed could be expected to result in a favourable long-term QOL with upfront osimertinib. The optimal treatment combination or sequence of radiotherapy (WBRT or SRS) with osimertinib from the viewpoint of OS and long-term QOL should be investigated.

The final OS analysis data of FLAURA trial indicates that osimertinib is the preferred first-line therapy for NSCLC patients with EGFR exon 19 and 21 mutation regardless of CNS metastases rather than gefitinib or erlotinib monotherapy. However, there are still other options for first-line treatment, such as dacomitinib, afatinib, erlotinib plus bevacizumab, and gefitinib plus pemetrexed-based chemotherapy in the cases of NSCLC patients without brain metastases. No prospective trials have directly compared these optional treatments to osimertinib in first-line setting. In the FLAURA trial, patients treated with first-line osimertinib showed longer PFS (18.9 months vs. 10.2 months; HR = 0.46; 95% CI, 0.37-0.57; *p* < 0.001) and longer OS (38.6 months vs. 31.8 months; HR = 0.80; 95% CI, 0.64-1.00; *p* = 0.046) with similar safety profile compared to those treated with standard EGFR-TKIs, gefitinib or erlotinib [12, 33]. The second-generation EGFR-TKIs, afatinib and dacomitinib, also showed superior efficacy to first-generation EGFR-TKIs [34, 35]. Importantly, gefitinib combined with carboplatin plus pemetrexed showed a significant superior median PFS (20.9 vs. 11.9 months; HR = 0.49, *p* < 0.001) and median OS (50.9 vs. 38.8 months; HR = 0.722, *p* = 0.021) compared to gefitinib alone with an acceptable toxicity profile in a recent phase III NEJ 009 study [36]. Although the authors mentioned that the OS benefit requires further validation, an OS of over 50 months is a remarkable result. A recent network meta-analysis indicated that osimertinib and gefitinib plus pemetrexed-based chemotherapy were associated with the best PFS and OS benefits for advanced *EGFR*-mutated NSCLC [37].

It should be noted that the FLAURA trial included patients with NSCLC exclusively harbouring common *EGFR* mutations only (exon 19 deletion or exon 21 L858R point mutation). Although AURA extension, AURA 2, and AURA 3 included a small number of patients with uncommon EGFR mutations (*n* = 9, 8, and 11, respectively, each approximately below 5%) such as exon 20 insertions, L861Q, S768I, and G719X, the numbers are too small to derive any suggestions [9–11]. The data regarding the efficacy of available EGFR-TKIs against NSCLC with uncommon *EGFR* mutations is limited, as patients with uncommon mutations were excluded from most clinical trials. Patients with uncommon mutations are a heterogeneous group with different sensitivities to different EGFR-TKIs [38]. Afatinib and osimertinib have some evidence in NSCLC with uncommon *EGFR* mutations. In a combined post hoc analysis of three prospective trials, LUX-Lung 2, LUX-Lung 3, and LUX-Lung 6, afatinib showed activity towards the most frequent uncommon *EGFR* mutations such as G719X, L861Q, and S768I, but not towards *de novo* T790M or exon 20 insertion mutations [39]. Additionally, second-generation EGFR-TKIs, especially afatinib, indicated broader activity across uncommon mutations compared to first- and third-generation EGFR-TKIs in preclinical studies [38, 40]. Some retrospective data also support the efficacy of afatinib [38, 41]. Recently, a first prospective multicentre phase II trial demonstrated osimertinib activity towards uncommon *EGFR* mutations. The CNS response rate was reported as 40% (two of five patients), with a complete CNS response in a patient harbouring the G719X mutation [42]. As both prospective analyses of afatinib and osimertinib included limited numbers of patients (*n* = 100 and 36, respectively), conclusions about CNS activity cannot be drawn and additional studies are needed [39, 42].

In conclusion, this case demonstrated that osimertinib could result in the complete remission of multiple brain metastases (as many as twenty lesions) of *EGFR*-mutated NSCLC without radiation therapy when used as first-line treatment. Moreover, radiation therapy for brain metastases can be deferred or even withheld especially when osimertinib is used as first-line treatment, because complete CNS remission can be occasionally achieved as early as one month even in patients with as many as twenty lesions. First-line treatment with osimertinib is the better treatment of choice compared with conventional treatment in cases of *EGFR*-mutated NSCLC with CNS metastases. A new treatment strategy for *EGFR*-mutated NSCLC with CNS metastases should be investigated with this potent EGFR-TKI, osimertinib, especially regarding the optimal combination or sequence of radiotherapy. Further large clinical studies are needed in this regard.
